# A Bedside Manual of Physical Diagnosis

**Published:** 1842-10

**Authors:** 


ART. XII.—*A Bedside Manual of Physical Diagnosis.* Second Edition. By CHARLES COWAN, M.D., Physician to the Reading Hospital.—*London*, 1842. 12mo, pp. 99.

WE noticed the first edition of this little work with much commendation, (Vol. III. p. 184.) It is considerably improved in its present form, yet not enlarged beyond its proper sphere of a pocket or bedside helper. It ought to be in the possession of all young auscultators.

